# Biportal Endoscopic Posterior Open‐Window Focal Debridement and Lavage for Sacroiliac Joint Tuberculosis

**DOI:** 10.1002/atn2.70199

**Published:** 2026-06-30

**Authors:** Wei Cheng, Wei Zhang, Hao Pan, Gaoyong Jia

**Affiliations:** ^1^ Department of Orthopaedics Hangzhou Traditional Chinese Medicine Hospital Affiliated to Zhejiang Chinese Medical University Hangzhou China

## Abstract

Sacroiliac joint tuberculosis (SJT), although relatively uncommon, may lead to severe destruction and functional impairment of the sacroiliac joint. Surgical intervention is recommended for patients with significant joint destruction, mild cystic changes, or sacroiliac joint instability. Early bone fusion may be facilitated through drainage of purulent fluid, curettage of necrotic tissue, and arthrodesis. Conventional surgery demands extensive osteotomy to create a sufficiently large bone window within the sacroiliac joint cavity for clearing infected tissue. The purpose of this technical note is to describe the details of biportal endoscopic iliac fenestration debridement for the management of sacroiliac tuberculosis.

## Graphical abstract

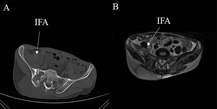

**VIDEO 1:** The patient is placed in the lateral decubitus position with the affected side up. The endoscopic working channel is determined at 1 cm lateral to the puncture localization needle. Under the endoscope, radiofrequency was used to clean the soft tissues on the surface of the iliac crest and expose the positioning of the Kirschner wire. A 3‐mm diamond burr was used to remove part of the iliac crest along the Kirschner wire, and the window was opened to reach the diseased area of the sacroiliac joint. The nucleus pulposus forceps removed the case‐like necrotic tissue and part of the necrotic bone, the Kirschner wire was removed, and radiofrequency was used to stop bleeding and ensure clear vision. After necrotic tissue was completely removed with nucleus pulposus forceps or laminectomy rongeurs, blood on the surface of healthy bone tissue was observed. Streptomycin was implanted into the diseased area of the sacroiliac joint with cannula, and an irrigation tube was placed. Video content can be viewed at https://doi.org/10.1002/atn2.70199.

Patients with sacroiliac tuberculosis presenting severe joint destruction, mild cystic changes, or sacroiliac joint instability are advised to undergo surgical intervention. Early bone fusion may be facilitated through drainage of purulent fluid, removal of necrotic tissue, and arthrodesis procedures.^1^ Current surgical approaches for sacroiliac joint tuberculosis (SJT) arthritis primarily comprise anterior, posterior, and combined anterior‐posterior approaches. Anterior debridement and bone grafting carry potential risks of adjacent structure injury, whereas the posterior approach offers a safer operative pathway.^2^ However, posterior surgery necessitates iliac windowing for lesion clearance, making complete debridement challenging to ensure. Biportal endoscopy provides a minimally invasive approach to the SJT. This endoscopic approach has been applied in the management of lumbar spinal tuberculosis and cement and lost reamer removal in revision hip arthroplasty.^3^ The purpose of this technical note is to describe the details of biportal endoscopic focal debridement and lavage for the management of SJT. It is indicated in patients with predominant posterior sacroiliac joint destruction with or without a posterior abscess or sinus tract (Table 1).

**TABLE 1:** Indications and Contraindications of Biportal Endoscopic Iliac Fenestration Debridement for the Management of Sacroiliac Tuberculosis

Indications	Contraindications
1. Predominant posterior sacroiliac joint destruction with or without a posterior abscess or sinus tract	1. Requires anterior support using the retroperitoneal approach 2. Requires internal fixation after lesion removal

## SURGICAL TECHNIQUE

### Preoperative Assessment

A patient diagnosed with SJT infection requires immediate quadruple antituberculosis drug treatment. Once symptoms such as body temperature and erythrocyte sedimentation are under control, aggressive nutritional support treatment is required. Antituberculosis drugs are used for approximately 2 weeks before the operation (Video 1).

**FIGURE 1:**
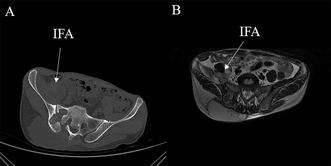
Biportal endoscopic debridement and lavage for sacroiliac joint tuberculosis. (A) The computed tomography axial image shows bone destruction in the right sacroiliac joint. (B) The magnetic resonance (MR) sagittal image shows anterior iliopsoas abscess and posterior sacroiliac joint abscess. (IFA, iliac fossa abscess.)

**FIGURE 2:**
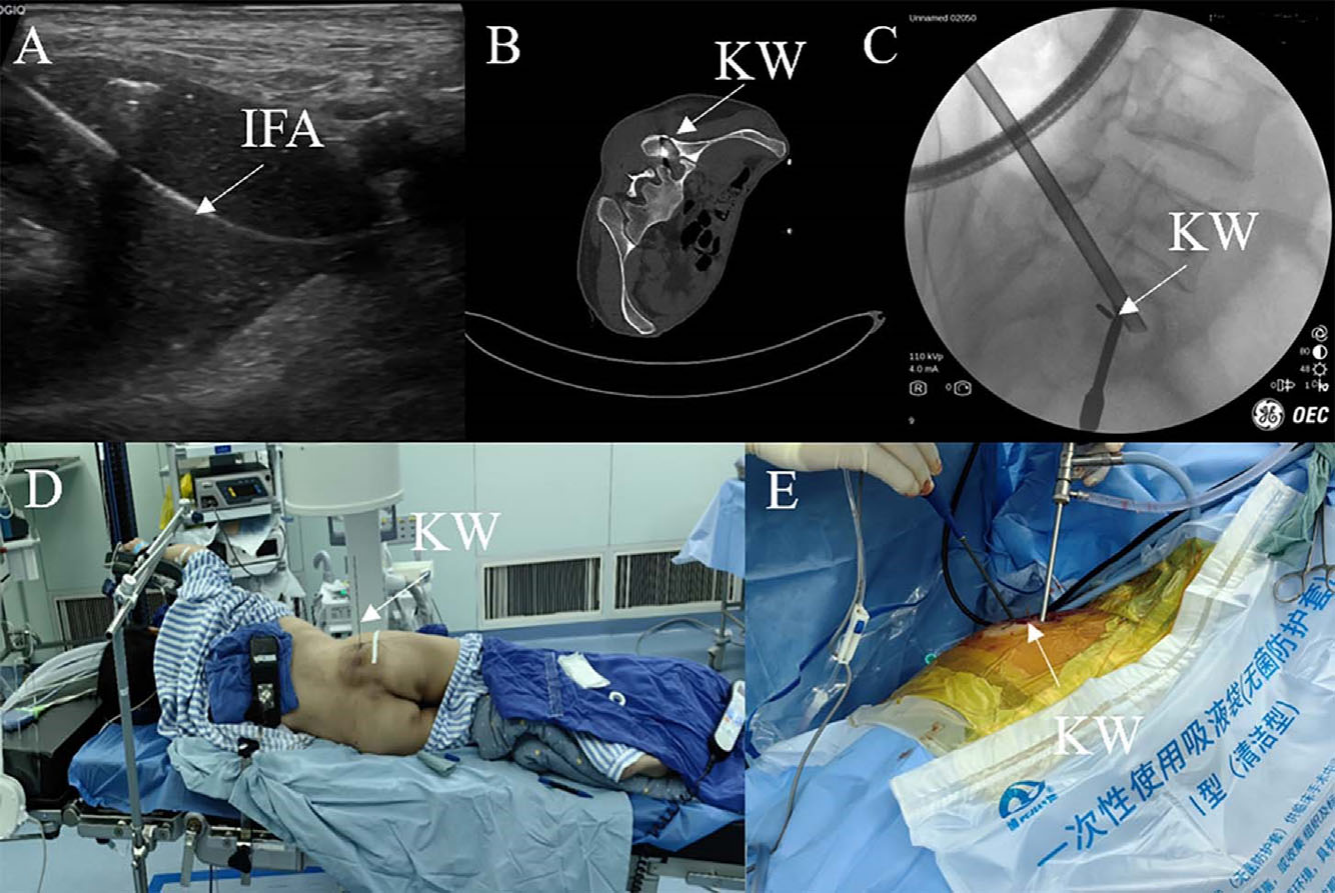
Biportal endoscopic debridement and lavage for sacroiliac joint tuberculosis. (A) Abscess puncture and drainage of anterior iliopsoas muscle guided by B‐ultrasound. (B) Computed tomography‐guided location of sacroiliac joint lesions. (C) The viewing and the working portals for the sacroiliac joint lesions. (D) The patient was placed in a lateral position. (E) Independent viewing and working portals were used to manage the lesions. (IFA, iliac fossa abscess; KW, Kirschner wire.)

Preoperative computed tomography (CT) and magnetic resonance imaging of the patient are carefully read to determine the size of bone destruction and the extent of abscess (Figure 1). B‐ultrasound‐guided puncture of the iliac fossa is conducted to drain the anterior pus (Figure 2A). The preoperative location is determined by multislice spiral CT, the puncture level of the lesion is determined, disinfection and tissue placement are performed, local anesthesia is administered with 0.5% lidocaine, Kirschner wire is inserted into the lesion under the guidance of CT, and the patient is sent to the operating room in a flat car. Figure 2B shows intraoperative CT‐guided puncture localization.

### Surgical Instruments

Surgical instruments used during the operation include a sterile saline irrigation system, a 30° endoscope, a high‐speed burr (Guizhou Zirui Technology, China), conventional arthroscopic facilities, a tool kit of radiofrequency systems (Jiangsu BONSS Medical Technology, China), and open spine surgical equipment, such as pituitary forceps and Kerrison rongeurs.

### Position and Creation of Portals

The patient is placed in a lateral position on a radiolucent operating table, and the procedure is performed under general anesthesia (Figure 2D). The viewing portal is made at the proximal end, 1 cm, and the working portal at the distal end, 1 cm, with the preoperative positioning guide needle as the center. A series of dilators and a cannulated working sleeve are inserted sequentially into the target site (Figure 2C). Separate viewing and working portals are created (Table 2).

**TABLE 2:** Pearls and Pitfalls of Biportal Endoscopic Debridement and Lavage for Sacroiliac Joint Tuberculosis

Pearls	Pitfalls
1. Preoperative computed tomography‐guided localization of lesions is essential for surgical planning 2. To avoid injury to the superior cluneal nerves, we must separate the soft tissues using progressive dilation of the trocar sheath when establishing the viewing and working portals	1. Peritoneal effusion due to inadvertent penetration of the sacrum with a drill

### Biportal Endoscopic Tissue Biopsy and Removal

**FIGURE 3:**
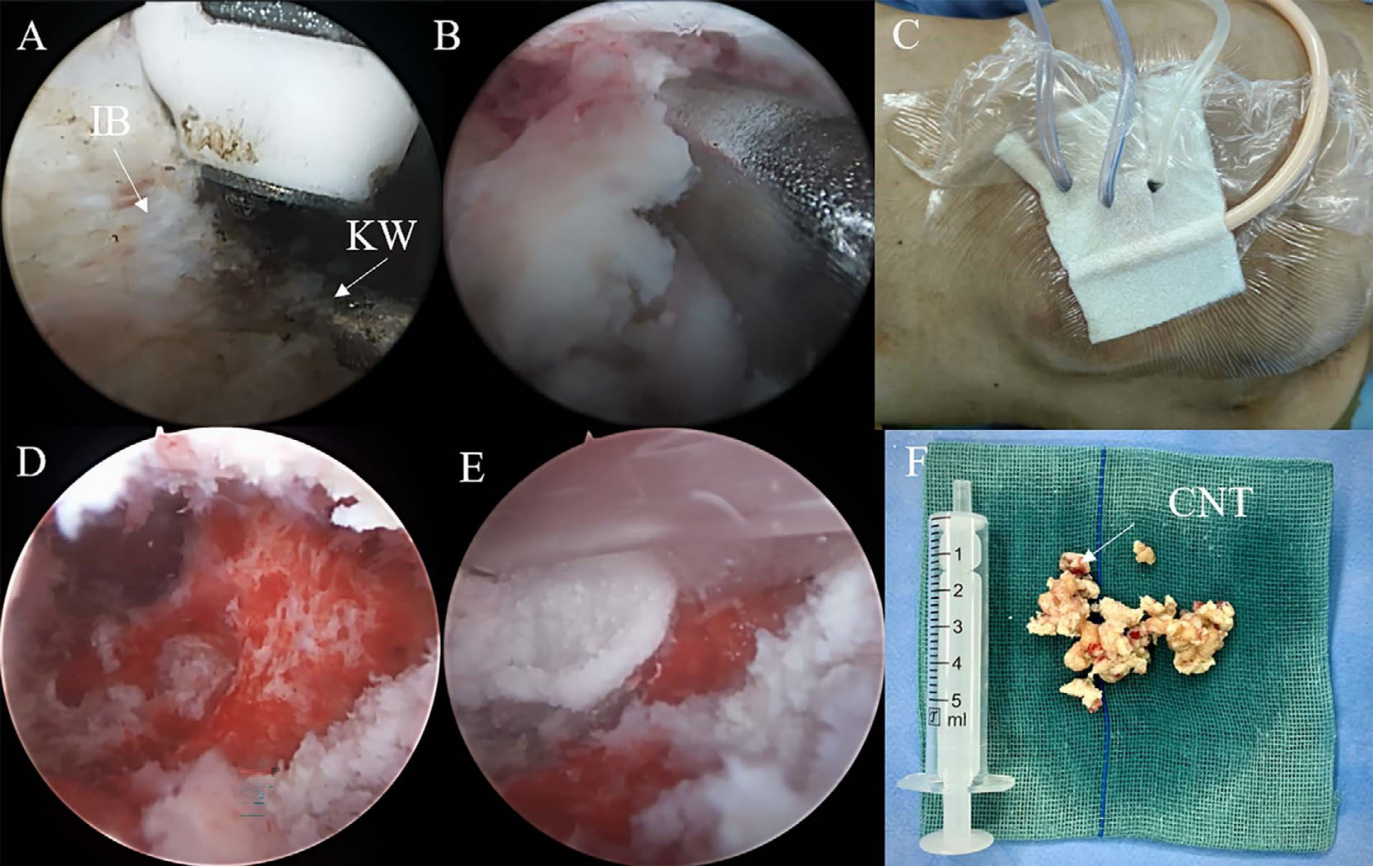
Biportal endoscopic debridement and lavage for sacroiliac joint tuberculosis. (A) The Kirschner wire was exposed using radiofrequency. (B) The endoscopic image of the biopsy and tuberculoma removal. (C) Postoperative placement of an irrigation tube in the surgical incision. (D) Necrotic bone tissue and caseous necrotic tissue were cleared. (E) Lesions were covered with streptomycin powder. (F) Cheesy necrotic tissue. (CNT, caseous necrosis tissue; IB, iliac bone; KW, Kirschner wire.)

Radiofrequency is used to clean the soft tissue, expose the iliac bone and Kirschner wire (Figure 3A), remove part of the bone along the Kirschner wire to reach the lesion area, pull out the Kirschner wire, and scrape the caseous necrotic tissue (Figure 3B,F). The focus of tuberculosis is completely curetted through the healthy bleeding bone (Figure 3D). The sacroiliac joint is irrigated with saline and dusted with 1.0 g of streptomycin powder (Figure 3E). Finally, 3 catheters are inserted into the affected lesion through 3 separate sheaths, with 1 catheter connected to a 2000‐mL saline solution bag for continuous irrigation and drainage.

### Postoperative Course

**FIGURE 4:**
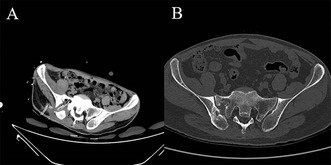
Biportal endoscopic debridement and lavage for sacroiliac joint tuberculosis. (A) Postoperative computed tomographic scan revealing the iliac window site following surgery, with the irrigation tube positioned within the sacroiliac joint lesion area. (B) At 1.5 years postoperatively, localized bone callus formation is evident within the sacroiliac joint lesion area.

Postoperative CT and magnetic resonance imaging showed that the abscess was completely cleared and the irrigating tube was located in the focal area (Figure 4A). At the 1.5‐year follow‐up, the patient's SJT lesion was found to be completely cleared, with fresh local bone callus formation (Figure 4B).

## DISCUSSION

The objective of surgery for tuberculous sacroiliitis is to remove the tuberculous lesion and fuse the sacroiliac joint.^1^ Traditional surgical approaches are categorized as anterior or posterior. Anterior surgery is primarily indicated for patients presenting with predominant anterior destruction of the sacroiliac joint; however, due to the presence of major vascular structures and the lumbosacral plexus within the pelvic cavity, the procedure is technically challenging and may be associated with increased blood loss. Posterior approaches offer safety and ease of manipulation, although dissection of the posterior sacroiliac ligament and joint capsule may compromise ligamentous stability around the joint.^2^ The transiliac approach is currently reserved primarily for iliac mass excision, its limitations being the inability to achieve complete debridement under full direct visualization and the restricted surgical space.^4^

In recent years, with the development of endoscopic technology, unilateral biportal endoscopy (UBE) technology is not only suitable for lumbar spinal stenosis but also reported for the treatment of spinal tuberculosis^5^ and tibial osteomyelitis^6^ owing to its advantages of flexible operation, wide field of vision, and complete debridement (Table 3).

**TABLE 3:** Advantages and Risks of Biportal Endoscopic Open‐Window Focal Debridement and Lavage for Sacroiliac Joint Tuberculosis

Advantages	Risks
1. Minimal incision 2. Minimal soft tissue damage 3. Thorough debridement of sacroiliac joint abscesses	1. Peritoneal effusion following sacral bone perforation 2. Incomplete removal of caseous necrotic tissue 3. Iatrogenic fracture

The advantages of this technique include minimal incision, minimal soft tissue damage, and thorough debridement of sacroiliac joint abscesses. Potential risks include peritoneal effusion following sacral bone perforation and incomplete removal of caseous necrotic tissue. In addition, adequate, combined, standardized, and long‐term postoperative antituberculosis chemotherapy is indispensable and important.

## DISCLOSURES

The authors (W.C., W.Z., H.P., G.J.) declare that they have no known competing financial interests or personal relationships that could have appeared to influence the work reported in this paper.

## FUNDING

This work was supported by the Key Discipline of Traditional Chinese Medicine in Zhejiang Province (2024‐XK‐57) and the Construction Fund of Key Medical Discipline of Hangzhou (2025HZZD16).

## ETHICAL APPROVAL AND INFORMED CONSENT

All procedures performed in studies involving human participants were in accordance with the ethical standards of the Ethics Committee of Hangzhou TCM Hospital and with the 1964 Helsinki Declaration and its later amendments or comparable ethical standards. Informed consent was obtained from all individual participants included in the study.
